# Influence of congruency and social episodic memory on subsequent social decision-making

**DOI:** 10.3758/s13421-025-01773-2

**Published:** 2025-08-13

**Authors:** A. M. Sklenar, A. N. Frankenstein, P. Urban Levy, E. D. Leshikar

**Affiliations:** Department of Psychology, University of Illinois Chicago, 1007 West Harrison Street (M/C 285), Chicago, IL 60607 USA

**Keywords:** Congruency, Impression formation, Impression memory, Social episodic memory, Social cognition, Decision-making

## Abstract

Research shows strong impacts of congruency on memory for social information, but whether memory advantages emerge for congruent or incongruent information is inconsistent. Social targets can have congruency between their facial expression (e.g., smiling, frowning) and behaviors (e.g., helping, hurting). The current study investigated the impact of congruency between valence of facial expressions and behaviors on memory and approach/avoidance (AA) decisions. At encoding (i.e., impression formation), participants formed positive or negative impressions of social targets. Social targets were represented by a picture with a positive or negative facial expression and a congruent or incongruent positive or negative behavior. At retrieval, we measured memory for multiple details (impressions, behaviors, facial expression) associated with targets encountered during encoding (impression formation). In a final approach/avoidance phase of the experiment, participants then judged whether they would approach or avoid social targets based on what they remembered about targets. Results showed that impression memory and behavior memory affected subsequent AA decisions, with correct memory for positive and negative impressions leading to approach and avoidance decisions, respectively. However, there was no impact of expression memory on AA decisions, suggesting participants did not base their decisions on irrelevant expression information. Further, results showed no effect of congruency on impression memory, behavior memory, or AA decisions, and limited impact on expression memory. Overall, findings may cast doubt on congruency/incongruency effects found in prior memory-related work, possibly suggesting an impact of the task.

Various types of information about social targets can be used when making inferences and decisions about other people. Behaviors and beliefs are important influencers of impressions of (Falvello et al., 2015; Leshikar & Gutchess, 2015; Leshikar et al., 2015a, b; Sklenar et al., 2023; Todorov & Uleman, 2003) and interactions with other people (Kadwe et al., 2022; Sklenar et al., 2021, 2022), because people spontaneously use information about what people have done to make inferences about the person’s traits and character (Todorov & Uleman, 2003). Although past behaviors may be the best predictors of how someone will behave in the future and therefore whether it would be good or bad to interact with someone, there are undoubtedly other types of information that influence impressions and subsequent decisions to approach or avoid others. Research shows that people pay great attention to visual information, are able to quickly process faces and the emotions they convey (Adams et al., 2006; de Jong et al., 2009) and often form impressions based on the face alone (Bzdok et al., 2011; Cassidy et al., 2012; Willis & Todorov, 2006). For example, based on facial structure alone, people make inferences about whether social targets are aggressive, competent, likeable, and trustworthy (Willis & Todorov, 2006). Facial expressions (e.g., whether someone is smiling or frowning, scowling, or fearful) have also been shown to be readily attended to, likely because expressions are perceived to convey intentionality (Adams et al., 2006; Lundqvist & Ohman, 2005; Öhman, 2009). Importantly, visual and behavioral information about a social target can be incongruent (inconsistent) with each other. Not only do people use visual information, they often do so at the expense of more meaningful behavioral information when the two types of information are incongruent (Olivola & Todorov, 2010). For example, Olivola and Todorov (2010) showed that when presented with a picture and information about social targets, people tend to rely more on the visual information from the face, which in turn made them less accurate in inferring characteristics of those targets than if they relied more on the verbal, descriptive information.

Congruency of information has a strong impact on memory. However, in the area of social episodic memory, the effect of congruency is not consistent. For example, past work investigating the impact of congruency between appearance-based inferences (e.g., perceived trustworthiness: Cassidy & Gutchess, 2015; Rule et al., 2012, Exp. 3; Suzuki & Suga, 2010; perceived dominance: Cassidy et al., 2012) and behavior on memory for social targets have found mixed results. Some studies have found that memory for congruent information was better than memory for incongruent information (Cassidy & Gutchess, 2015; Cassidy et al., 2012; Rule et al., 2012, Exp. 3). This congruency advantage seemingly occurs because congruent information is more easily incorporated into one’s schema for the person, and also potentially because guessing is more common when processing demands are high, and guessing tends to favor congruent information (Stangor & McMillan, 1992). For example, Cassidy and Gutchess (2015) and Rule et al. (2012, Exp. 3) both presented trustworthy and untrustworthy looking faces (and neutral for Cassidy & Gutchess, 2015) paired with congruent or incongruent trustworthy or untrustworthy behaviors, and later tested different types of memory. Rule et al. (2012, Exp. 3) found a congruency advantage within untrustworthy targets, where congruent untrustworthy targets (targets with untrustworthy faces and untrustworthy behaviors) were recognized better than incongruent untrustworthy targets (targets with trustworthy faces and untrustworthy behaviors). Cassidy and Gutchess (2015) also found a congruency advantage in memory for the behavior-based impression previously formed of the target, with better impression memory for congruent than incongruent trials, and for both congruent and incongruent than neutral trials. However, unlike Rule et al. (2012, Exp. 3), Cassidy and Gutchess’s (2015) congruency advantage was driven by trustworthy trials: congruent trustworthy targets (trustworthy face and trustworthy behavior) were remembered better than incongruent trustworthy targets (untrustworthy face and trustworthy behavior). These studies finding congruency advantages are often limited to certain situations, and there are no clear reasons for when the congruency advantage is found and when it is not. However, some research suggests that congruency advantages increase with increasing task difficulty Cassidy et al. (2012; Exp. 2).

Unlike the congruency advantage described above, other studies have found better memory for incongruent (e.g., a trustworthy face with an untrustworthy behavior) than congruent information (untrustworthy face with untrustworthy behavior; Stangor & McMillan, 1992; Suzuki & Suga, 2010). This incongruency advantage seemingly occurs because the incongruent information is more salient and requires more effort to be incorporated into one’s schema for a person. Therefore, this increased effort and processing might make the incongruent memory more likely to be remembered. For example, Suzuki and Suga (2010) had participants play a game with trustworthy-looking and untrustworthy-looking targets who were either good, bad, or neutral lenders. Suzuki and Suga found an incongruency advantage within untrustworthy opponents, with better memory for bad lenders who looked trustworthy than for bad lenders who looked untrustworthy. Similar to the discussion on congruency advantages, incongruency advantages are also often limited to certain situations, as other researchers have shown (Bell et al., 2012; Kroneisen et al., 2021). Regardless of finding congruency or incongruency advantages, these studies showcase that appearance-based information has a strong impact, biasing decisions made about others (Cassidy & Gutchess, 2015; Cassidy et al., 2012). However, in many studies on congruency between facial features and behaviors, there are two factors that might influence the results rather than congruency itself: violation of expectancy and the availability of appearance-based information at retrieval.

One component of past congruency work that might influence results rather than congruency itself is that incongruency has often been conflated with a violation of expectancy. Studies typically present two pieces of congruent or incongruent information at different times: an initial piece of information, and then a later piece of congruent information that is consistent with the initial information, or incongruent information that is at odds with the initial information. Thus, in these studies, the incongruent information violates participants’ expectations based on the initial information (Stangor & McMillan, 1992). In this scenario where incongruency equates to a violation of an expectation from earlier information, it is possible that the task design itself might induce a congruency or incongruency advantage. For example, Cassidy and Gutchess (2015), Cassidy et al. (2012), and Suzuki and Suga (2010) each presented a picture of the targets’ face first, allowing targets to make inferences from the face about the person’s trustworthiness or dominance. Later, both studies presented a behavior performed by the target that was either congruent or incongruent with the prior inference. Thus, the initial inference from the visual information (face) was a mere guess as to whether the person was trustworthy or dominant based on facial stereotypes. The later verbal information about the person’s behavior was the more relevant and meaningful information to be prioritized for the task of remembering whether the person was trustworthy or dominant. Therefore, in these and similar studies, the more meaningful behavioral information (which was important to do well on the task of remembering whether the person was trustworthy or dominant) was always the information presented later. Thus, an incongruent trial meant there was a violation of their expectation based on the initial impression (i.e., their initial assumption based on the face was wrong). In this experimental setup, incongruency advantages are more beneficial to update their impression of the person and avoid harm when interacting with them in the future, but congruency effects may be found because of the demand of updating so much information. Therefore, it is possible in these prior studies that the congruent/incongruent advantages are not a reflection of greater attention needed to process and update the incongruent information or easier processing of congruent information, but rather that the advantage reflects the demands of the task itself. In this study, we investigate the impact of congruency on memory and memory-based decisions while reducing potential for violation of expectancy.

In the current study, we wanted to include stimuli that made it possible to present both pieces of incongruent information simultaneously, so as to not require an updating of a prior expectancy, and also so that both pieces of incongruent information make sense together (e.g., scenarios where it makes sense for a positive facial expression to accompany a negative behavior so that the conflicting valences do not feel at odds). Therefore, we investigate the influence of congruency between facial expression valence (positive/happy, negative/angry) and behavior valence (positive, negative) on social episodic memory (i.e., memory for specific details associated with past encounters with targets) as well as subsequent decisions to approach or avoid social targets. Importantly, opposing valences of behaviors and facial expressions are not necessarily a violation of prior information. Whereas happy expressions often convey friendliness and angry faces often convey a threat, in real life, it is not always so simple. There are many complex scenarios where someone could have a happy expression while performing a negative behavior, or an angry expression while performing a positive behavior. Unlike prior inferences, facial expressions and behaviors can be incongruent based on valence (e.g., positive face with a negative behavior) but still make sense in the same context. For example, if a shifty salesman lies to make a sale, he may be smiling either to convince the customer or because he is happy his deception worked. Therefore, the current study can examine incongruency under conditions less likely to induce expectancy violations (e.g., some initial information about targets is incongruent with later information about targets). This allows investigation of the impact of congruency (between more meaningful behaviors and more salient facial expressions) without a violation of a prior expectation. In the current study, there is no inherent advantage to prioritizing congruent or incongruent information since there is no opportunity to update information about targets. Neither a congruency nor incongruency advantage in memory would be beneficial for accuracy in the current study, so this investigation has the potential to uncover whether and why a congruency or incongruency advantage emerges while reducing the potential influence of expectancy violations.

The second component of past congruency work that might influence results rather than congruency itself pertains to the availability of information from faces at retrieval. The appearance-based information in each of these prior studies on face-behavior congruency was participants’ perceptions of a target’s trustworthiness or dominance based on the target face itself (Cassidy & Gutchess, 2015; Cassidy et al., 2012; Rule et al., 2012, Exp. 3; Suzuki & Suga, 2010). Therefore, because the target face was presented at both encoding and retrieval, the perception of trustworthiness or dominance from the face was available both at encoding and at retrieval, whereas the behavioral information was only available at retrieval if participants correctly remembered the behavior. It is possible that the strong impact of appearance-based information in these studies was simply because participants forgot the behavioral information and had to base their decision on the only information that was available to them at retrieval: the information that was readily available from the person’s face. If this were the case, then participants could just re-form the appearance-based trustworthiness or dominance decision at retrieval instead of using memory for the behavior or initial impression. Therefore, in the current study, we were interested in investigating the impact of congruency on memory for social targets when the facial information is memory-based rather than present at retrieval. This way, both the information from faces and behaviors would be memory-based at retrieval, and we could better test the impact of congruency of facial and behavior valence. Unlike perceptions based on facial features, facial expressions and the emotions conveyed by them are dissociable from the faces expressing them (Hartley et al., 2015). Using facial expressions and stimuli that do not require violation of a previous expectation allow us to better investigate whether memory for social targets is influenced by congruency of facial expression valence and behavior valence. Thus, in the current investigation, we use happy and angry facial expressions at encoding (impression formation), but neutral expressions at retrieval so that neither the valenced information from faces nor behaviors is available at retrieval. Doing so allows for a more rigorous test of congruency on memory and memory-based social decision-making (e.g., approach/avoidance decisions).

In addition to investigating the impact of congruency on memory, we also investigate how congruency’s impact on memory affects subsequent decisions to approach or avoid social targets. Approach/avoidance (AA) decisions have long been studied in social psychology, resulting in the identification of myriad social factors that influence AA decisions. These social factors include readily available information such as the physical features of social targets (e.g., emotional expressions, Adams et al., 2006; Chen & Bargh, 1999; and perceived trustworthiness of a person’s face, Oosterhof & Todorov, 2008; Todorov, 2008), motivational factors (e.g., motivational influences of prevention and promotion focus; Higgins, 1997, 1998), and group-level information (e.g., race, religion, gender, age; Kidwell & Booth, 1977; Parrillo & Donoghue, 2005; Smith & Dempsey, 1983; Weaver, 2008). Recent research has shown that memory also impacts AA decisions (Kadwe et al., 2022; Murty et al., 2016; Schaper et al., 2019; Sklenar et al., 2021, 2022). Of the recent work finding evidence of memory’s effect on AA decisions, two studies used monetary games to show that memory for whether opponents are cheaters or cooperators influences participants’ decision to interact with them on future games (Murty et al., 2016; Schaper et al., 2019). More recently, Sklenar et al. (2021, 2022) and Kadwe et al. (2022) tested whether memory for impressions previously formed of social targets influenced later AA decisions to want those targets as their neighbors. In these studies, targets were represented either by a picture (Sklenar et al., 2021, 2022) or a name (Kadwe et al., 2022) along with information about the target (trait-implying behavior performed by the target; Kadwe et al., 2022; Sklenar et al., 2021; and sometimes a political-ideological label [conservative or liberal; Sklenar et al., 2021, Exp. 2]; or political ideological beliefs, Sklenar et al., 2022). In the encoding (impression formation) phase, participants formed positive or negative impression of social targets. Then in the memory (retrieval) phase, participants indicated if they remembered forming a positive or negative impression of targets (or if the target was a previously unseen new target), and answered questions about memory for other information associated with targets. Last, in the approach/avoidance phase, the pictures or names of each target were presented and participants decided whether they would want each target to be their neighbor based on memory. Results across a multitude of experiments show that correct memory for impressions based on different types of information (wide range of trait-implying behaviors and political ideologies) strongly impact decisions to approach or avoid others. More specifically, Sklenar et al. (2021, 2022) and Kadwe et al. (2022) each found that when participants correctly remembered the impressions previously formed of social targets, memory for positive impressions led to approach decisions and memory for negative impressions led to avoidance decisions. Thus, research consistently shows that social episodic memory for a variety of details such as whether someone is a cheater or cooperator (Kroneisen et al., 2021; Murty et al., 2016; Schaper et al., 2019), and memory for impressions based on a range of trait characteristics (Kadwe et al., 2022; Sklenar et al., 2021) and ideological beliefs (Sklenar et al., 2021, 2022) influences decisions to approach or avoid others. However, more work is needed to understand the extent and ways that different types of memories influence AA decisions. The current study adds to this work by investigating whether the congruency of behavior valence and facial expression valence influence the impact of memory on AA decisions.

In summary, this work investigates the impact of congruency between facial expression valence and behavior valence on memory (particularly impression memory) and the influence of facial expression/behavior valence congruency and memory on subsequent AA decisions. In this experiment, participants first form impressions of social targets represented by a picture of a face with a happy or angry expression paired with a positive or negative trait-implying behavior. Then participants complete a memory test for the impression, the behavior, and the expression of the target. We focus on memory for self-generated information (i.e., impressions), in part, because prior work shows that self-generated information tends to be well-remembered (McCurdy et al., 2017, 2019, 2021, McCurdy, Sklenar et al., 2020a, McCurdy, Viechtbauer et al., 2020b; McCurdy & Leshikar 2022). Finally, participants make AA decisions by deciding whether they would want each target as a neighbor. We make four predictions in this investigation: the first about memory effects, and the last three about the effects of memory on subsequent AA decisions. For the effect on memory, we planned to run an Initial Impression × Congruency analysis of variance (ANOVA) for each of the three types of memory: impression, behavior, and expression. First, we hypothesized that there would be a main effect of congruency for each of the three different types of memory, with better impression memory, behavior memory, and expression memory for congruent than incongruent trials (Hypothesis 1). Hypothesis 1 would be consistent with the congruency advantage found by Cassidy and Gutchess (2015), Cassidy et al. (2012), and Rule et al. (2012, Exp. 3), and consistent with the idea that without a violation of expectancy, congruent information would be easier to remember, even more so when the conflicting facial information is not present at retrieval. An alternative possibility, however, is that there is no congruency nor incongruency advantage in memory, if the congruency/incongruency effects in past work were driven by task demands due to violation of expectancy rather than how congruent/incongruent information was processed. Second, we expected that when impression memory is correct, positive impressions and positive behaviors, but not positive expressions would be associated with approach decisions (Hypothesis 2). Although it is possible that facial expressions could still affect AA decisions, we expect that in the absence of valenced facial information after encoding, participants will rely on behaviors and impressions when making AA decisions given that behaviors and impressions should be more meaningful predictors of future behavior. Third, and analogous to Hypothesis 2, we expected that when impression memory is correct, negative impressions and negative behaviors, but not negative expressions would be associated with avoidance decisions (Hypothesis 3). Hypotheses 2 and 3 are consistent with our prior work showing that accurate memory for valenced information, such as impressions and behaviors, strongly impacts subsequent approach or avoidance decisions (Kadwe et al., 2022; Sklenar et al., 2021, 2022). Fourth, turning to the impact of memory accuracy and congruency on AA decisions, we focus on match percentage, which is the percentage of AA decisions that matched the initial impression (i.e., approaching targets previously associated with positive impressions; avoiding targets previously associated with negative impressions); as opposed to when the AA decision does not match the initial impression (i.e., approaching targets previously associated with negative impressions; avoiding targets previously associated with positive impressions). We expected a main effect of memory accuracy, with greater match percentage when impression memory was correct, when behavior memory was correct, and when expression memory was correct than when incorrect (Hypothesis 4). Said differently, we predict in Hypothesis 4 that participants should be less likely to make the expected AA decision when their memory is incorrect than when it is correct. We do not have strong predictions for the impact of congruency on AA decisions given the novel nature of this work. However, we again see two likely possibilities: either a congruency effect from removing the direct influence of facial information at retrieval/the AA decision, or no congruency effect if the (congruency) effects in past work were due to the nature of the experimental task used in prior studies, and not on congruency per se. Overall, results of this investigation will yield better understanding on factors that influence social episodic memory, and how such memory representations affect subsequent social decision-making (i.e., AA decisions).

## Method

### Participants

A total of 58 participants took part in the study, but data was excluded from 10 participants. One participant was under 18 years old and could not be included in analyses per the Institutional Review Board, and nine were excluded from analyses for having unusable data.^1^ Thus, the remaining participants included 48 undergraduates (33 women, 15 men; ages 18–29 years,* M* = 19.42, *SD* = 2.18; Latino 29%, White 27%, Asian 25%, Black 15%, Other 2%) from the University of Illinois Chicago. Power analyses using the R package *WebPower* indicated that detection of a large effect (*f* = 0.5; for an Initial Impression × Congruity interaction) with a power of .8 and an alpha of .05 would require a sample size of 45. Participants were recruited through the university’s subject pool, provided informed consent in accordance with the university’s Institutional Review Board, and received course credit for their participation.

### Stimuli

Stimuli included pictures of 72 faces, including 48 from the Chicago Face Database (Ma et al., 2015) and 24 from the NimStim Face Set (Tottenham et al., 2009), which were used as social targets. Half of the faces were female and half male, and half were Black and half White. Each face had three different expressions: neutral, angry, and happy. The Chicago Database Face set had multiple versions of some expressions, so only the closed-mouth versions were selected to be consistent with the faces selected from the NimStim Face Set. Both databases depicted targets wearing a white shirt, with no jewelry, on a white background, with the face tightly cropped. Stimuli also included 72 behavioral sentences that were either positive or negative in valence,^2^ but were designed to make sense with either a positive or negative expression (e.g., “This person yelled at the boy for teasing his classmates.” would make sense with a positive expression because this is a positive behavior implying the target is a considerate, but it could also make sense with a negative expression because this person expressed anger).^3^ Face and sentence stimuli were both counterbalanced to appear during encoding or as novel items in the memory test (retrieval phase). Faces were further counterbalanced to appear with either positive or negative sentences, and sentences were counterbalanced to appear with positive or negative expressions. Faces were also counterbalanced to have a positive or negative expression at encoding. The study was presented using E-Prime software (Psychology Software Tools, Pittsburgh, PA, USA), with text (in white, 30-point Arial font) and images presented against a black background.

### Procedure

The experiment consisted of three phases: encoding (i.e., impression formation), retrieval, and approach/avoidance, which took place in a single session. Before starting the experiment, participants were trained on both the encoding (impression formation) phase as well as the retrieval phase. Because participants were trained on both the encoding and retrieval phases, this means participants were aware of the upcoming memory test, making this an intentional encoding procedure.

**Fig. 1:**
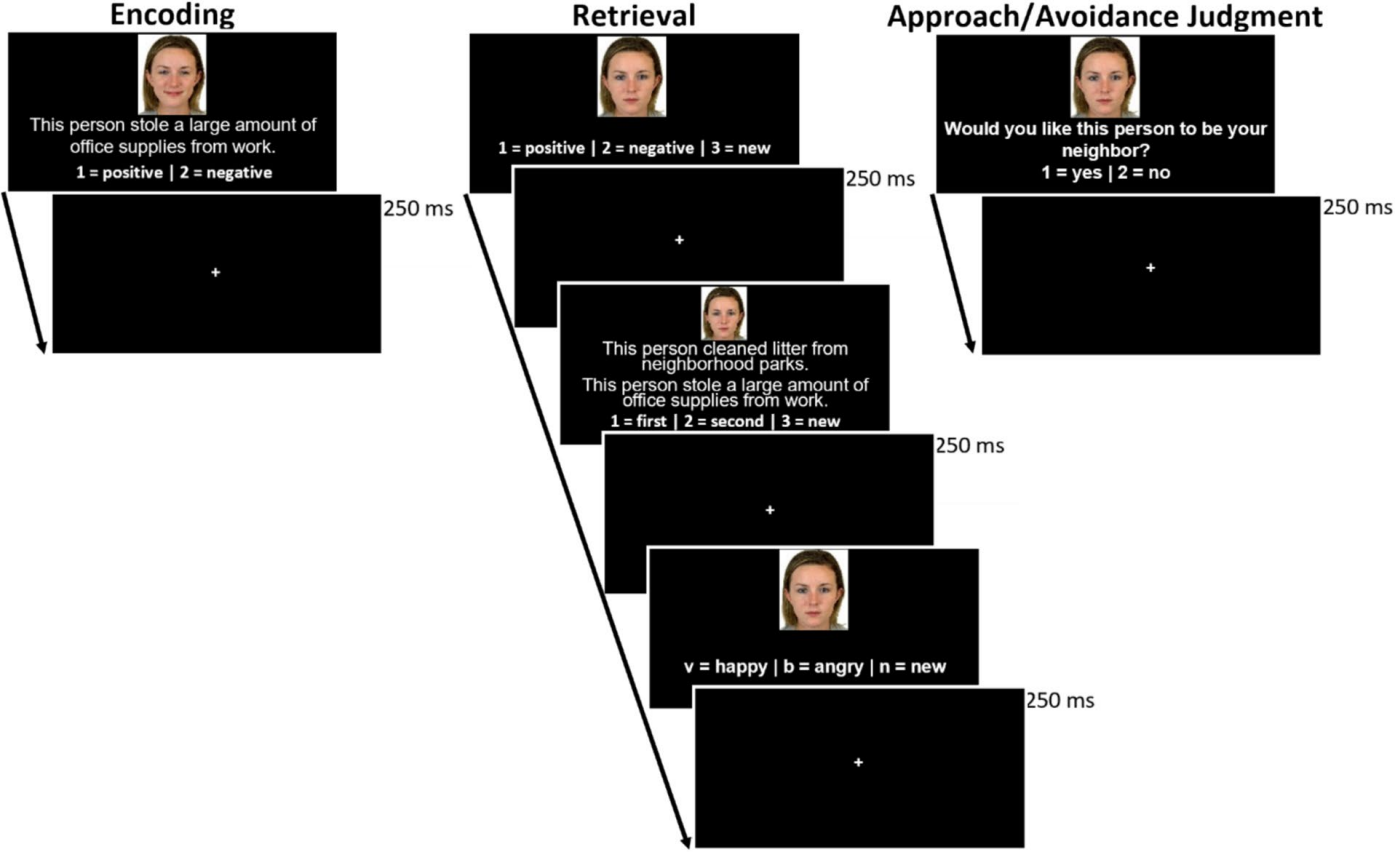
Procedure of encoding phase, retrieval phase, and approach/avoidance judgment phase of the experiment

There were two blocks each of encoding and retrieval, and encoding and retrieval blocks were interleaved with no distractor task in between. Each block of encoding consisted of 24 trials (resulting in 48 encoding trials total across the two blocks). In each encoding phase trial, a picture of the target’s face with either an angry or happy expression was presented along with either a positive or negative behavioral sentence below the picture (see Fig. 1). Participants were asked to use their own personal judgment to form a positive or negative impression of each target based on the face and the behavior, and to indicate their impression by key press (v = positive | b = negative). Trials were self-paced and separated by a fixation cross presented for 250 ms. Trials were pseudorandom, such that no more than four male or female targets or four Black or White targets and no more than four positive or negative behaviors or four positive or negative expressions were presented consecutively.

After completing an encoding block, participants immediately started the associated retrieval block. Each block of retrieval consisted of 36 self-paced trials: 24 “old” faces that were presented at encoding, and 12 “new” targets (resulting in a total of 48 “old” trials and 24 “new” trials across both blocks which yields 72 retrieval trials overall). For each trial, participants made three memory judgments (impression memory, behavior memory, expression memory). First, participants were shown a picture of a target with a neutral expression and were asked to remember which impression they had formed of that target during encoding or if the target was new (v = positive | b = negative | n = new).^4^ Second, participants were shown two sentences, one positive and one negative, along with the neutral face and asked to indicate which of the two sentences was previously presented with that face at encoding or if the target (i.e., the person) was new (v = first sentence | b = second sentence | b = new). Trials were pseudorandomized so that half of the “old” trials (i.e., those seen during encoding) had the correct sentence first, and half of the “old” trials had the correct sentence second. Thus, behavior memory assessed which behavior was presented with that specific face, and not just whether behaviors had been previously seen. Third, participants were asked which expression was on the targets’ face at encoding, or if the target was new (v = happy | b = angry | n = new). In addition to the pseudorandom order used in the encoding phase, retrieval trials were further pseudorandomized so that no more than four old or new trials and no more than four trials where the first sentence was correct or incorrect were presented in a row.

After completing two blocks of both encoding and retrieval, participants then completed the approach/avoidance (AA) phase of the experiment. Each of the 72 targets from the previous phases were presented with neutral expressions. For each self-paced trial of the AA measure, participants decided whether they would want that person to be their neighbor (v = yes | b = no). Participants were asked to base their decision on their memory for that target, or to go with their intuition in the case that they could not remember. Finally, participants completed a demographic questionnaire, and were debriefed and granted credit for participating.

## Results

In this section, we report responses generated at encoding. Following that, we then report our memory analyses, and then our analysis for the approach/avoidance data. Starting first with responses generated at encoding (i.e., impression formation), participants formed positive impressions for 49% (*SE* = .01) of trials, and they formed negative impressions for 51% of trials. This distribution of impressions is in line with our use of half positive and half negative information associated with targets.

**Fig. 2:**
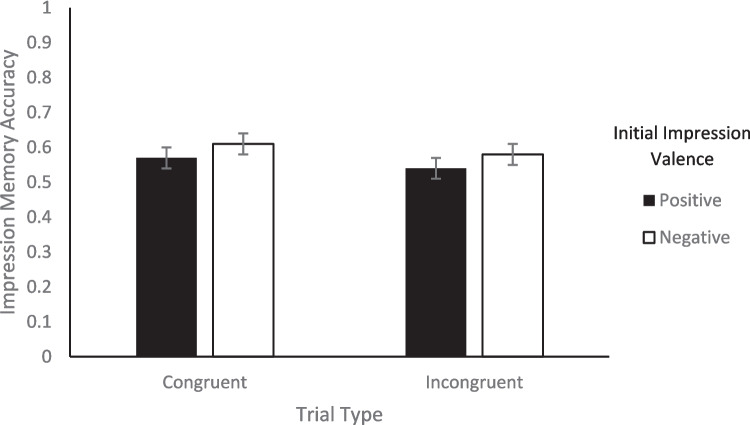
Percentage of correct impression memory responses as a function of valence of initial impression and congruency between behavior valence and expression valence. *Note.* Error bars represent standard error of the mean

**Fig. 3:**
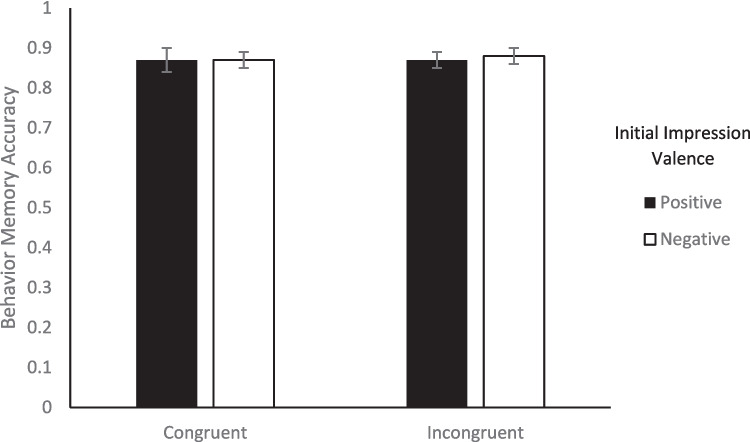
Percentage of correct behavior memory responses as a function of valence of initial impression and congruency between behavior valence and expression valence. *Note.* Error bars represent standard error of the mean

**Fig. 4:**
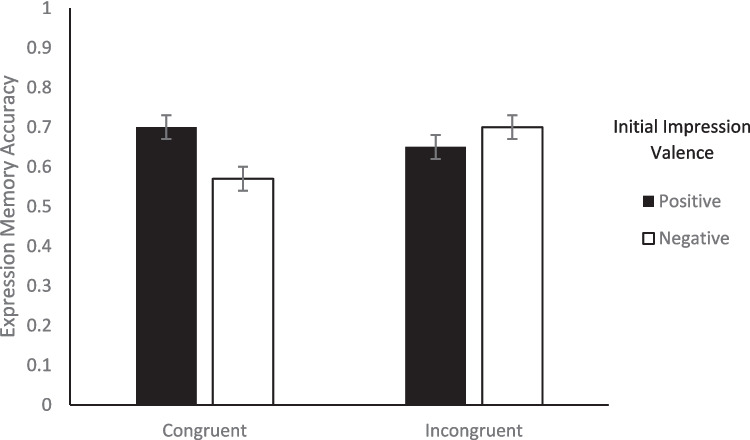
Percentage of correct expression memory responses as a function of valence of initial impression and congruency between behavior valence and expression valence. *Note.* Error bars represent standard error of the mean

Turning to memory, we looked at the effect of congruency between facial expression valence and behavior valence on memory to evaluate Hypothesis 1. To test whether there was a difference in memory accuracy between congruent and incongruent trials, we conducted a 2 (Initial Impression: positive, negative) × 2 (Congruency: congruent, incongruent) repeated-measures ANOVA on the percentage of correct memory responses for each of the three types of memory. For impression memory, none of the main effects nor the interaction was significant (*F*s < 1.28, *p*s > .265; see Fig. 2).^5^ For behavior memory, none of the main effects or the interaction was significant (*F*s < 0.09, *p*s > .782; see Fig. 3). For expression memory, there was a significant initial impression x congruency interaction, *F*(1, 47) = 6.00, *p* =.018, η_p_^2^ = 0.11. Within congruent trial types, expression memory was significantly better for positive impressions (*M* = 0.70, *SE* = 0.03) than negative impressions (*M* = 0.57, *SE* = 0.03), *t*(47) = 2.77, *p* = .039, *d* = .43, but within incongruent trial types, there was no difference between positive (*M* = 0.65, *SE* = 0.03) and negative impressions (*M* = 0.70, *SE* = 0.03), *t*(47) = 1.26, *p* = .591, *d* = .18. Neither of the main effects were significant (*F*s < 2.66, *p*s > .110). Therefore, congruency between facial expression valence and behavior valence had minimal impact on memory in the current study, only affecting expression memory (see Fig. 4). These results are consistent with the alternative possibility for Hypothesis 1.

**Fig. 5:**
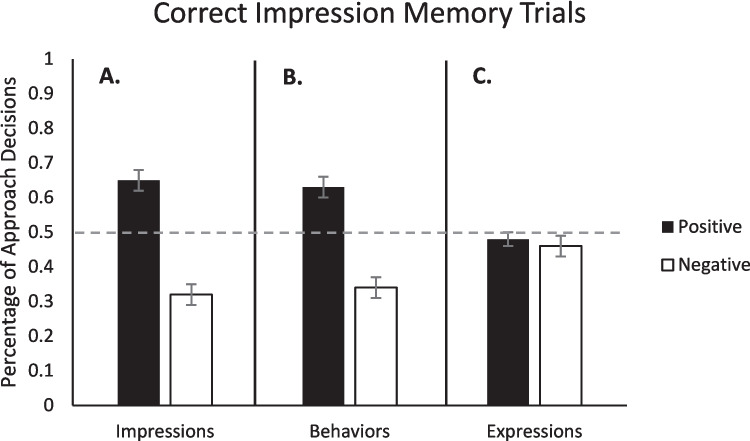
Percentage of approach decisions as a function of valence of initial impression (**A.**), valence of behaviors (**B.**), and valence of expressions (**C.**) for trials where impressions were correctly remembered compared to a 50% baseline

We next examined approach/avoidance responses for the correctly remembered old trials. To evaluate Hypotheses 2 and 3, we conducted a standard one sample *t*-tests comparing the percentage of “yes” (approach) responses for positive and negative impressions to a 50% baseline, as in prior work (Kadwe et al., 2022; Sklenar et al., 2021, 2022). A 50% baseline represents the percentage of AA trials that participants would choose to approach new, never-before-seen targets.^6^ On trials where impression memory was correct, the percentage of “yes” responses was significantly greater than baseline for targets associated with positive impressions (*M* = 0.65, *SE* = 0.03),* t*(47) = 5.24, *p* < .001, *d* = 0.76, and significantly less than baseline for targets associated with negative impressions (*M* = 0.32, *SE* = 0.03), *t*(47) = −6.55, *p* < .001, *d* = 0.95 (see Fig. 5A). Thus, memory influenced AA decisions. We ran similar comparisons based on valence of the behaviors and the expressions, in place of impressions, which showed that on trials where impression memory was correct, the percentage of “yes” responses was significantly greater than baseline for targets who performed positive behaviors (*M* = 0.63, *SE* = 0.03),* t*(47) = 4.65, *p* < .001, *d* = 0.67, and significantly less than baseline for targets that performed negative behaviors (*M* = 0.29, *SE* = 0.03), *t*(47) = −5.66, *p* < .001, *d* = 0.82 (see Fig. 5B). Taken together, the impression and behavior memory results suggest memory for various details (impressions, behaviors) strongly influence subsequent AA decisions. There was no difference, however, in the percentage of “yes” responses between targets who had positive expressions and baseline (*M* = 0.48, *SE* = 0.02),* t*(47) = −0.63, *p* = .529, *d* = 0.09, or between targets who had negative expressions and baseline (*M* = 0.46, *SE* = 0.03),* t*(47) = −1.33, *p* = .189, *d* = 0.19 (see Fig. 5C).^7^

**Fig. 6:**
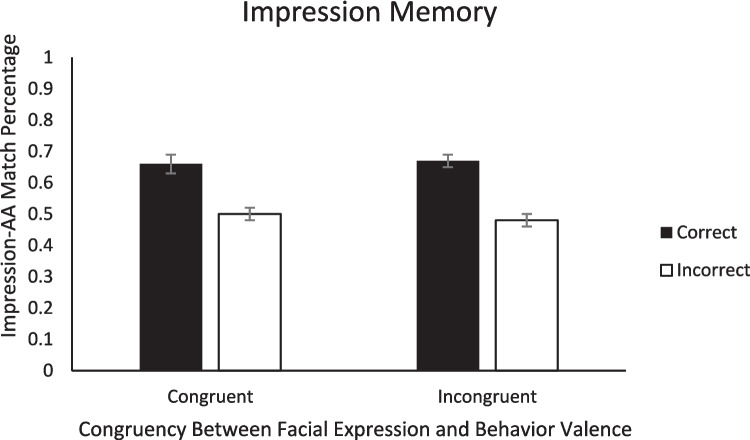
Percentage of match decisions (when the AA decision matched the initial impression) as a function of impression memory accuracy and congruency between behavior valence and expression valence. *Note.* Error bars represent standard error of the mean

**Fig. 7:**
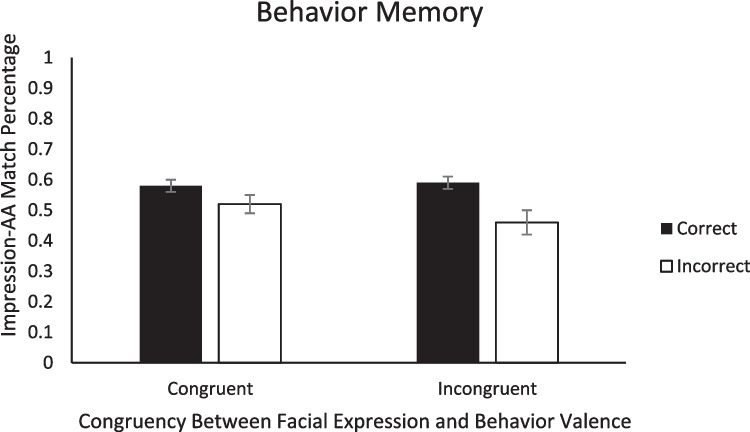
Percentage of match decisions (when the AA decision matched the initial impression) as a function of behavior memory accuracy and congruency between behavior valence and expression valence. *Note.* Error bars represent standard error of the mean

**Fig. 8:**
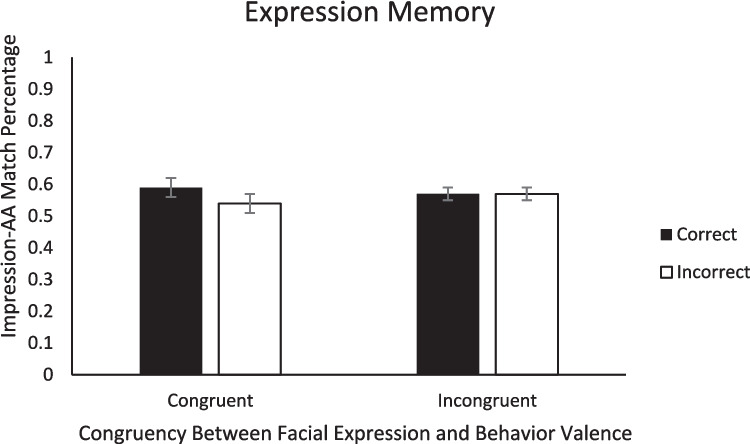
Percentage of match decisions (when the AA decision matched the initial impression) as a function of expression memory accuracy and congruency between behavior valence and expression valence. *Note.* Error bars represent standard error of the mean

Last, to evaluate Hypothesis 4, we conducted 2 (Congruency: congruent, incongruent) × 2 (Memory Accuracy: correct, incorrect) ANOVAs on the percentage of matched responses (positive impression ➔ approach decision; negative impression ➔ avoidance decision) within each of the three types of memory. First, for impression memory, there was a main effect of impression memory accuracy, with a higher match percentage when impression memory was correct (*M* = .67, *SE* = .02) than when impression memory was incorrect (*M* = .50, *SE* = .01), *F*(1, 47) = 50.73, *p* < .001, η_p_^2^ = .52. Because we found higher match percentage for the correct relative to incorrect trials, this result further demonstrates that memory influences subsequent AA decisions. Neither the main effect of congruency nor the Congruency × Impression Memory Accuracy interaction were significant (*F*s < 0.46, *p*s > .506; see Fig. 6). For behavior memory, there was also a main effect of behavior memory accuracy, with more matches when behavior memory was correct (*M* = .58, *SE* = .02) than when behavior memory was incorrect (*M* = .49, *SE* = .02), *F*(1,46) = 8.68, *p* = .005, η_p_^2^ = .16, which again is consistent with the role of memory in social decision-making. Neither the main effect of congruency nor the Congruency × Behavior Memory Accuracy interaction were significant (*F*s < 1.40, *p*s > .244; see Fig. 7). Last, for expression memory, none of the main effects or interactions were significant, (*F*s < 1.20, *p*s > .242; see Fig. 8).

## Discussion

The goal of the current study was to assess the extent congruency between valence of facial expressions and behaviors affected memory for social targets, and further, how memory accuracy for social targets affected subsequent decisions to approach or avoid social targets. There were three main findings for our four hypotheses. First, we found limited support that congruency between facial expression and behaviors affected social episodic memory (Hypothesis 1). Such findings may suggest that past congruency effects were driven by experimental procedures (that may have introduced expectancy violations). Second, we found that affective positive social episodic memory representations (impressions, behaviors) increased approach decisions (Hypothesis 2), whereas negative social episodic memory representations (impressions, behaviors) increased avoidance decisions (Hypothesis 3). Third, we further examined the effects of memory on subsequent AA decision by focusing on match percentage (i.e., approaching targets previously associated with positive impressions; avoiding targets previously associated with negative impressions). Our results showed greater match percentage for the correctly remembered compared to incorrectly remembered trials (for impression memory and behavior memory, but not expression memory) which largely supports Hypothesis 4, and further underscores the role of memory in social decision-making.

First, looking at the effect of congruency on memory, we found no significant effects of congruency on impression memory, behavior memory, or expression memory. Therefore, Hypothesis 1 was not supported. There was an Initial Impression × Congruency interaction for expression memory, whereby expression memory was better for positive than negative impressions within congruent trials only. It is unclear why expression memory was worse for negative than positive impressions on congruent trials. When there are valenced memory effects in younger adults, memory is often better for negative than for positive information (Liberzon et al., 2003; Lundqvist & Ohman, 2005; Ogawa & Suzuki, 2004). However, memory is sometimes better for positive information in social contexts, especially for gist-level impressions (Leshikar, et al., 2015a, b; Leshikar & Gutchess, 2015; Sklenar et al., 2022, 2023). It may be the case that memory for facial expressions on congruent trials were more gist-based, which is why we observed improved memory for positive information. Interestingly, some work suggests that smiling faces may be more memorable than other types of facial expressions (D’Argembeau & Van der Linden, 2004, 2011), and thus it may be that our findings of improved memory for the positive trials, at least under conditions face/behavior congruency, may be due in part to heightened memorability of smiling faces. More research is needed to see if this is a consistent effect, and if so, why it occurs. It is possible that the absence of a congruency effect on any of the three types of memory in the current study was due to removing violation of expectancy, which in turn removed the benefit of preferentially remembering congruent or incongruent information. Perhaps congruency/incongruency effects seen in the past are not a reflection of differences in how congruent and incongruent information are processed per se, but rather how congruent and incongruent information are processed based on their relative importance for the specific task at hand. This could suggest that it is not a congruency/incongruency effect, but more specifically a violation of expectancy effect, such that there needs to be a benefit or detriment to updating information to find an effect of congruency, as others have argued in the domain of social episodic memory (Bell et al., 2014; Kroneisen 2018; Kroneisen & Bell 2022). Understanding factors that influence memory is an important area of work (Bell et al., 2015a, b; Fejzic et al., 2025; Giannakopoulos et al., 2021, 2024; Leach et al., 2019; Leshikar et al., 2010, 2012, 2017; Matzen et al., 2015; Meyers et al., 2020; Mieth et al., 2021), and the current investigation adds to that body of work.

In this investigation we found limited evidence that congruency affected social episodic memory. We made this observation under experimental conditions where congruent/incongruent information about targets was presented simultaneously, which is a departure from prior work where congruent/incongruent information was shown at separate times (e.g., an initial piece of information is congruent/incongruent with later information about specific targets). Interestingly, some work in the 1980s focused on “outcome dependency” when examining congruency effects in social episodic memory. Outcome dependency is the idea that participants will process information, particularly incongruent information, about social targets differently based on what they are expected to do with that information later. For example, one investigation had participants learn congruent and incongruent information about a social target, and then were asked to work with that social target on a creative task to compete for a cash prize (Erber & Fiske, 1984). In one condition (the outcome dependency condition), participants were told their work with the social target would be evaluated together for the cash prize, and in the other condition (non-outcome dependency), participants were told their work did not depend on the other social target. Results showed that memory for the consistent information about the social target was not affected by outcome dependency; however, memory for inconsistent information was higher in the outcome dependency condition than in the non-outcome dependency, which suggests that participants were more attentive (and better remembered) incongruent information about targets, when they knew they would have to work closely with the target subsequently. Turning back to the current investigation, although participants did not know about the subsequent AA decisions, they did know that their memory (impression, behavior, expression) would be evaluated for social targets. Given this, it is possible that our use of intentional encoding procedures (where participants knew about the memory test), may have affected our memory effects, particularly for incongruent information about targets. Future work in this area should further investigate memory for facial/behavior congruency effects under incidental encoding conditions where participants are unaware of the memory test.

Second, for the effect of social episodic memory on AA decisions, we compared the percentage of yes decisions to the neighbor question for positive and negative impressions, behaviors, and expressions to a 50% baseline within trials where memory was correct. Results showed that when impression memory was correct, positive impressions and positive behaviors, but not positive expressions led to approach decisions, supporting Hypothesis 2 and consistent with prior work (Kadwe et al., 2022; Murty et al., 2016; Schaper et al., 2019; Sklenar et al., 2021, 2022). Similarly, when impression memory was correct, negative impressions and negative behaviors, but not negative expressions, led to avoidance decisions, again consistent with prior work (Kadwe et al., 2022; Murty et al., 2016; Schaper et al., 2019; Sklenar et al., 2021, 2022) and supporting Hypothesis 3. These results provide continued support for the impact of social episodic memory on AA decisions but suggest that expression memory is not relied on when making AA decisions because it is not a meaningful predictor of decisions to approach or avoid others when more relevant information is available. In the current study, it would be more beneficial to rely on memory for the behavior or impression than the facial expression, given that the trait-implying behavior is a better predictor of future behaviors, and the facial expression was only congruent with one’s behavior half the time. One possibility is that participants were ignoring the facial expression at encoding, however this seems unlikely, as expression memory accuracy was not only better than chance, but actually better than impression memory accuracy (although lower than behavior memory accuracy). It seems more likely that participants, whether intentionally or unintentionally, placed less emphasis on memory for the expression valence when making the AA decision and instead relied on their memory for the more meaningful behaviors and impressions. This result may be surprising given how strongly influential facial information has been in prior work, even when more meaningful behavioral information was available (Bzdok et al., 2011; Cassidy et al., 2012; Olivola & Todorov, 2010; Willis & Todorov, 2006). However, past work has tended to use appearance-based information that was available both at encoding and at retrieval, as perceptions of a person’s trustworthiness or dominance come from the person’s face itself (Cassidy & Gutchess, 2015; Cassidy et al., 2012; Suzuki & Suga, 2010). Thus, unlike behavioral information that had to be remembered, facial information in prior work was available during the memory test. Therefore, the current study could suggest that the overreliance on facial information at the expense of more relevant behavioral information found in the past may be more the result of having that facial information readily available at the time of the memory test. When both the facial and behavioral information need to be remembered, it seems that people are less likely to over-rely on the facial information. Exploring social cognitive processes is an important empirical pursuit (Banaji & Crowder, 1989; Burden et al., 2021; Cassidy et al., 2013; Colton et al., 2013; Green et al., 2008; Ilenikhena et al., 2021; Jackson et al., 2019; Leshikar & Duarte, 2012, 2014; Leshikar et al., 2015a, b, 2016; Sedikides & Green, 2009), and the current investigation adds to that scientific goal.

Third, we investigated the impact of memory accuracy and congruency on match percentage for each of the three memory types. We found the expected greater match percentage for correct than incorrect memory trials both for impression memory accuracy and for behavior memory accuracy, but not for expression memory accuracy, thus partially supporting Hypothesis 4. In combination with the comparison to baseline findings described earlier (Hypotheses 2 & 3), this last set of findings provide additional support for the impact of memory for behaviors and expressions on AA decisions, while suggesting that expression memory does not as strongly influence subsequent AA decisions. Therefore, when more meaningful predictors of decisions to approach or avoid are available (such as behaviors), participants may be able to ignore their memories for less meaningful facial information, despite how influential facial information can be (Bzdok et al., 2011; Cassidy et al., 2012; Olivola & Todorov, 2010; Willis & Todorov, 2006). Interestingly, although we used a variety of different behaviors in this investigation (that could fit with positive or negative facial expressions), it may be that some types of behaviors may be more meaningful, or better predictors, of future behaviors of targets which in turn could even more strongly affect subsequent social decisions. For example, some types of behavioral information, such as whether a person is a cheater or a helper (e.g., prosocial) may be more important and impactful in making subsequent AA decisions. Such a finding would be in line with prior work showing that memory can be especially good for cheaters (Buchner et al., 2009; Kroneisen, 2018; Suzuki & Suga, 2010) or for those engaging in prosocial behaviors (Urban Levy et al., 2023). Thus, future work in this area should investigate how different types of behaviors that are known to be especially memorable might affect subsequent AA decisions. There was no impact of congruency on AA decisions, which could suggest that congruency does not have a strong influence over subsequent AA decisions about social targets. However, given the memory finding in the current study (i.e., generally no congruency effects), this AA finding may further reflect the possibility that congruency/incongruency effects in prior work were not actually inherent congruent/incongruent effects, and instead might have resulted from features of the experimental design (e.g., violation expectancy; available facial information at retrieval). More research will be important to understanding the mechanisms contributing to the impact of congruency on memory and social decisions and when these effects occur. Overall, our AA data showed that social episodic memory is used in social decisions, which adds to a larger body of work showing what and how memory is used adaptively in daily life (Bell et al., 2015a, b; Frankenstein et al., 2020, 2022, 2025; Murty et al., 2016; Nairne, 2010; Patel et al., 2022; Udeogu et al., 2022; Villasenor et al., 2021; Wong et al., 2017; Wood et al., 2024).

In this investigation, we found clear evidence that social episodic memory representation strongly influenced subsequent social decisions (Hypotheses 2–4). Interestingly, some work examining the relationship between memory and social decisions has found evidence of guessing while making social decisions. For example, in one investigation using an economic game task, participants showed a bias to presume untrustworthy looking targets were cheaters, and trustworthy looking targets were cooperators, while making decisions about how much to invest with such targets (Kroneisen et al., 2021). Such results suggest that participants are prone to guessing biases when making social decisions. Although biases undoubtedly manifest in social decision tasks, we were careful in this investigation to only examine social decisions for targets where we knew participants could remember details about those same targets (correct memory). As a larger point, and as we argue elsewhere (Leshikar, 2024; Sklenar & Leshikar, 2025), we think to truly examine the relationship between social episodic memory and subsequent social decision-making, it is important to use an experimental approach that involves three phases: an initial encounter phase with targets (e.g., encoding), a retrieval phase where memory for specific targets is assessed, and a separate social decision-making stage where the participant make some judgment about specific targets. We think it is critical to only evaluate decisions for targets that participants accurately remember to truly examine the relationship between memory and decisions. Without knowing what (if anything) a participant can remember about specific social targets, it is more challenging to investigate the role of memory in social decision-making. Future work investigating the relationship between social episodic memory and social decisions should use this three-experimental-phase approach to better understand how people form and use memory representations while making critical social decisions.

Although we found limited effects of congruency on memory and strong evidence that social episodic memory representations (both positive and negative) affected AA decisions, there are two limitations to this investigation worth describing. First, we used face stimuli from two different face databases. Although we worked to use comparable faces, it is possible that the faces could have differed on dimensions such as attractiveness, trustworthiness, or baby-facedness which could have affected our data. We did perform a manipulation check and found that impression memory for faces from the NimStim set were similar to the faces from the Chicago Faces Database, which suggests that faces were largely comparable. Second, in our memory test at retrieval, we used twice as many “old” trials as “new” trials. We did this to balance the number of “correct” response choices for the impression memory decision (e.g., approximately 24 trials were associated with positive impression at encoding/impression formation, 24 trials were associated with negative impressions at encoding/impression formation, and 24 trials were “new” targets). It is possible, however, that our use of greater numbers of old relative to new trials could have affected our memory data. Future work in this area should use equal numbers of old and new trials at retrieval to further advance understanding of congruency effects in social episodic memory.

## Conclusion

Investigating various factors that influence decisions to approach or avoid others is important to build understanding of the cognitive and social-cognitive mechanisms underlying AA decisions, and the real-world impact of how and why we interact with others. We found no impact of congruency between valence of behaviors and facial expressions on memory or on AA decisions. These findings suggest a possible reinterpretation of past congruency work, where past incongruency advantages might not have been the result of greater attention and processing to update incongruent information, but rather that incongruency advantages might simply have been due to incongruent information being more important for the task. Similarly, past congruency advantages might not have been the result of the greater ease of processing congruent information, but rather that congruent or incongruent visual information was just given prioritized (earlier) access in prior studies or the fact that some information was present at the time of retrieval. The current results could suggest that when congruency involves no violation of expectancy and the experimental task lends no benefit to having greater attention and memory for congruent or incongruent information, perhaps neither congruent nor incongruent information will be prioritized. Further, we continued to find support for the impact of social episodic memory for impressions and behaviors on subsequent AA decisions. Interestingly, however, we did not find the same effects for facial expressions, suggesting that people can rely on memory for more meaningful and relevant behaviors and impressions when making AA decisions. Thus, memory for salient physical features, like facial expressions, are not always overly relied upon. This finding may have important implications for interpretations of prior research, where it is possible that prior over-reliance on facial information might have been at least partially the result of the facial information being available both at encoding and at retrieval.

## Data Availability

Data are available upon reasonable request.

## Footnotes

1 These nine participants indicated that they remembered fewer than 10 of either positive or negative impressions in the memory phase. This is unlikely, indicating that they were not taking the task seriously, and further meant that there were not enough trials to perform the analyses. Of these nine, three said they remembered only nine negative impressions, two remembered only seven negative impressions, one said they remembered only seven positive impressions, one remembered only three positive and one negative impression and almost exclusively said targets were new, one said they remembered only one positive impression, and one said they remembered only one negative impression.

2 Prior to this investigation, we normed these behavior stimuli. To do so, we started with an initial pool of 144 behavior statements. We recruited an independent sample of participants to rate each of the behaviors on four dimensions: valence, arousal, fit with positive or negative facial expressions (e.g., the extent the behavior “fit” with a target smiling or frowning while performing that behavior), and typicality (e.g., how common or typical is the behavior). From this initial pool of 144 behaviors, we selected the 72 behaviors we used in this investigation. Our final set of positive behaviors were rated as positive, and our set of negative behaviors was rated as negative, while both sets were equated on valence, fit with positive or negative facial expression, and typicality.

3 Similarly, “This person bragged that the IRS has not caught them for tax evasion.” could make sense with a negative expression because this is a negative behavior implying the target is a dishonest person, but it could also make sense with a positive expression.

4 At retrieval we used 48 old targets along with 24 new targets. The reason we did this is so that the number of responses for the first retrieval decision about impression memory would be balanced across these trial types, with 24 positive “old” trials, 24 negative “old” trials, and 24 new trials. If we had used 48 “new” faces and 48 “old” faces, this would have resulted in an imbalance in correct responses (e.g., more “new” responses than the other response choices for the impression memory decision).

5 Although we used faces from two different databases that depicted targets similarly (e.g., a white shirt, with no jewelry, on a white background, with the face tightly cropped), it is always possible that differences across databases could affect memory data. As a manipulation check, we examined impression memory accuracy for the NimStim and CFD faces separately. Across conditions and participants, impression memory accuracy was identical for the NimStim (*M* = .63, *SE* = .02) and the CFD faces (*M* = .63, *SE* = .02).

6 We have confirmed the validity of the 50% baseline in a pilot study with an independent sample of participants who made the the same approach/avoidance decision as in the current study, but in response to faces that had not been shown before and without behaviors. The percentage of approach decisions was exactly 50%, supporting our use of 50% as baseline. Additionally, because the analysis to examine Hypothesis 4 does not use the 50% baseline, it provides converging evidence of the effect of memory on AA decisions independent of a baseline.

7 In our primary analysis, we looked at the percentage of yes responses to that AA decision for the correctly remembered old trials. For the new targets (e.g., novel targets that were not seen at encoding), we can also examine the “yes” percentage for new trials correctly recognized as novel (i.e., correct rejections), as well as the trials novels falsely remembered as associated with positive impressions (i.e., positive false alarms) and negative impressions (i.e., negative false alarms). Although these trial types are not a focus of the current investigation, participants made “yes” responses for .53 (*SE* = .05) of the positive false alarm trials, .39 (*SE* = .05) of the negative false alarm trials, and .34 (*SE* = .03) of the correctly rejected trials.
